# Efficacy and safety of olezarsen in lowering apolipoprotein C-III and triglycerides in healthy Japanese Americans

**DOI:** 10.1186/s12944-024-02297-5

**Published:** 2024-10-03

**Authors:** Ewa Karwatowska-Prokopczuk, Anastasia Lesogor, Jing-He Yan, Angelika Hoenlinger, Alison Margolskee, Lu Li, Sotirios Tsimikas

**Affiliations:** 1 Ionis Pharmaceuticals, Carlsbad, CA USA; 2 Novartis Pharma AG, Basel, Switzerland; 3 Division of Cardiovascular Medicine, Department of Medicine, University of California San Diego, 9500 Gilman Drive, BSB 1080, La Jolla, CA 92093-0682 USA

**Keywords:** Triglycerides, ApoC-III, Ethnicity, Antisense oligonucleotide, Pancreatitis, Cardiovascular disease

## Abstract

**Background:**

Olezarsen is a GalNAc_3_-conjugated, hepatic-targeted antisense oligonucleotide that lowers apolipoprotein C-III (apoC-III) and triglyceride levels. The efficacy and safety of olezarsen has not previously been studied in ethnically diverse American populations. The aim of this study is to assess the effect of olezarsen in healthy Japanese Americans.

**Methods:**

A randomized, placebo-controlled, double-blind phase 1 study was performed in 28 healthy Japanese American participants treated with olezarsen in single-ascending doses (SAD; 30, 60, 90 mg) or multiple doses (MD; 60 mg every 4 weeks for 4 doses). The primary, secondary, and exploratory objectives were safety and tolerability, pharmacokinetics, and effects of olezarsen on fasting serum triglycerides and apoC-III, respectively.

**Results:**

There were 20 participants (16 active:4 placebo) in the SAD part of the study, and 8 participants (6 active:2 placebo) in the MD part of the study. For the primary endpoint, no serious adverse events or clinically relevant laboratory abnormalities were reported. The majority of olezarsen plasma exposure occurred within 24 h post-dose. In the SAD cohorts at Day 15 the percentage reduction in apoC-III/TG was − 39.4%/ − 17.8%, − 60.8%/ − 52.7%, and − 68.1%/ − 39.2% in the 30, 60 and 90 mg doses, respectively, vs 2.3%/44.5% increases in placebo. In the MD cohort, at Day 92 the percentage reduction in apoC-III/TG was − 81.6/ − 73.8% vs − 17.2/ − 40.8% reduction in placebo. Favorable changes were also present in VLDL-C, apoB and HDL-C.

**Conclusions:**

Single- and multiple-dose administration of olezarsen was safe, was well tolerated, and significantly reduced apoC-III and triglyceride levels in healthy Japanese Americans.

**Supplementary Information:**

The online version contains supplementary material available at 10.1186/s12944-024-02297-5.

## Introduction

Apolipoprotein C-III (apoC-III) is a glycoprotein secreted by the intestine and liver that binds to triglyceride-rich lipoproteins (TRLs), such as chylomicrons, very-low-density lipoprotein (VLDL), and their remnants [1, 2]. ApoC-III downregulates lipoprotein metabolism by inhibiting lipoprotein and hepatic lipases and by reducing hepatic uptake of TRLs via low-density lipoprotein (LDL) and LDL receptor-related protein receptors [1–3]. In 2008, it was observed that people with heterozygous loss-of-function mutations in *APOC3* had lower triglyceride concentrations and reduced risk of cardiovascular disease [4]. Subsequently, apoC-III has become a target of therapy for patients with familial chylomicronemia syndrome (FCS), with several studies leading to the approval of volanesorsen (Waylivra®) in the European Union for this indication[3, 5–7] and in Brazil for both FCS and familial partial lipodystrophy [8]. Additional developments in the field include the GalNAc_3_-conjugated hepatic-targeted antisense oligonucleotide (ASO) olezarsen [9, 10], and the GalNAc_3_-conjugated hepatic-targeted siRNA plozasiran (ARO-APOC3) [11]. Clinical studies are either complete or underway to assess the role of inhibiting *APOC3* messenger RNA for FCS [16], for severe hypertriglyceridemia (SHTG, defined as plasma triglyceride levels ≥ 500 mg/dL) [12], and for hypertriglyceridemia (plasma triglyceride levels of 150 to 499 mg/dL) with or without elevated cardiovascular risk [13]. Whereas FCS is primarily associated with pancreatitis, SHTG, and its association with elevated remnant cholesterol, is associated with both pancreatitis and cardiovascular disease [14, 15].

Previously published phase 1/2 and phase 2 studies showed that multiple doses of olezarsen reduced apoC-III by 66% to 89% at 3 months and by 30% to 74% at 6 months, depending on dosing, in subjects with triglycerides ≥ 90 mg/dL and triglycerides 200 to 500 mg/dL at baseline, respectively [9, 10]. Similarly, triglycerides in these studies were reduced by 61% to 71% and 23% to 60% at the same timepoints and doses, respectively. Olezarsen, dosed at 50 mg and 80 mg subcutaneously monthly, is being evaluated in phase 3 clinical trials for the reduction of apoC-III and triglycerides in patients with FCS and SHTG. The Balance (NCT04568434) trial for FCS enrolled 66 patients and recent data revealed significant reductions in triglycerides with the 80 mg olezarsen dose, and marked reductions in pancreatitis events [16]. The CORE (NCT05079919) and CORE-2 (NCT05552326) trials for severe hypertriglyceridemia (triglycerides > 500 mg/dL) will include over 1000 patients combined.

Prior studies of olezarsen included single doses ranging from 10 to 120 mg and multiple doses ranging from 10 to 50 mg monthly but included a low number of patients of diverse ethnicities [9, 10]. In more recent studies of olezarsen, 6 Asian patients were included in the Balance study and 1 Asian patient was included in the Bridge study [16, 17]. Small studies have shown similar apoC-III concentrations in healthy Japanese and White populations [18, 19], although apoC-III mutations associated with hypertriglyceridemia may be identical or differ between people of Japanese descent and White patients [20, 21]. Prior studies of olezarsen included few non-White subjects. This study evaluated the safety, pharmacokinetics and efficacy of single and multiple doses olezarsen in healthy Japanese American subjects to expand the experience in more ethnically diverse populations.

## Materials and methods

### Study population

Participants were healthy individuals in the United States, 18 to 65 years of age, of non-childbearing potential, and of Japanese descent (first-, second-, or third-generation Japanese; each set of parents qualifying as being of Japanese descent under the prior generation). Additional eligibility criteria included good health, normal vital signs, weight of ≥ 45 kg, body mass index ≤ 35.0 kg/m^2^, and serum triglyceride levels ≥ 90 mg/dL at screening.

### Study objectives and endpoints

The primary study objectives were to assess the safety and tolerability of olezarsen following single and multiple subcutaneous doses in healthy Japanese American participants. Safety and tolerability endpoints included adverse events (AEs), laboratory assessments related to safety, physical examination findings, vital signs, and electrocardiogram (ECG) findings. Secondary objectives were to assess the pharmacokinetics of olezarsen following single and multiple subcutaneous doses. Exploratory objectives were to determine the effect of olezarsen on apoC-III levels and fasting serum triglyceride levels based on relative changes from baseline.

### Study design

This was a randomized, placebo-controlled, participant- and investigator-blinded, single-ascending and multiple-dose phase 1 study conducted at 1 site (Parexel International, Early Phase Clinical Unit, Los Angeles, USA) in healthy Japanese American participants. A local Institutional Review Board approved the study, which was conducted in accordance with the Declaration of Helsinki, the Guidelines for the International Council for Harmonisation of Technical Requirements for Pharmaceuticals for Human Use Good Clinical Practice, and the Health Insurance Portability and Accountability Act of 1996. All participants provided written informed consent prior to any study-related activities.

Following a screening period of up to 28 days and a baseline period to confirm eligibility (Day − 1), 20 participants were randomized to receive a single dose of either subcutaneous olezarsen 30, 60, or 90 mg, or matched placebo, as described in Supplementary Table 1. The 30-mg and 60-mg single-ascending–dose cohorts of 6 participants were randomized 5:1 to olezarsen or placebo. The 90-mg cohort of 8 participants was randomized 6:2 to olezarsen or placebo. Participants remained domiciled at the study center for 48 h following dose administration and underwent safety, pharmacokinetic, and pharmacodynamic monitoring. Following discharge on Day 3, participants underwent outpatient monitoring, sampling, and/or evaluations on Days 8, 15, 30, 60, 90, 120, and 150 (end of study). Participants were required to use contraception during the study period until 13 weeks after the last study drug dose for cohorts 2, 4, and 6, and until 16 weeks after the last study drug dose for cohorts 1, 3, and 7. Dietary restrictions included avoiding consumption of grapefruit, grapefruit juice, Seville oranges, caffeine, excessive alcohol consumption, and tobacco products. Participants were required to fast for 10 h prior to dosing and follow-up visits, and strenuous activity was prohibited from 72 h before admission until discharge. The study subjects were expected to follow their usual dietary and activity habits during the study period.

Once the single-ascending–dose data were reviewed and found to be acceptable, the multiple-dose testing commenced, with screening and baseline periods similar to the single-dose cohort period. Following randomization of 8 participants 6:2 to either subcutaneous olezarsen 60 mg or matched placebo, participants received a dose every 4 weeks (Days 1, 29, 57, and 85) for a total of 4 doses. Participants were domiciled at the study center for 48 h after the first dose, as well as on Days 84, 85, and 86. Participants underwent monitoring, sampling, and/or evaluations on Days 8, 15, 29, 43, 57, 71, 85, 86, 87, 92, 99, 113, 127, 141, 176, and 204 (end of study). A consort diagram is in Supplementary Fig. 1.

### Assessments

Safety assessments included AEs, vital signs, 12-lead ECGs, physical examinations, and clinical laboratory assessments. AEs were coded with MedDRA version 21.0. Clinical laboratory assessments included clinical chemistry, coagulation, hematology, high sensitivity C-reactive protein, thyroid panel, total complement activity, routine and extended urinalysis, urine renal biomarkers, and viral serology. Renal function, liver chemistry, and platelet counts were monitored regularly to ensure they did not meet stopping criteria, which were specified in the protocol.

Pharmacokinetic assessments were based on venous blood and urine samples and measured concentrations of olezarsen in plasma and urine. Plasma samples were analyzed using a validated hybridization-based assay with electrochemiluminescence (ECL) detection. Urine samples were analyzed using a qualified hybridization enzyme-linked immunosorbent assay. Pharmacokinetic samples were analyzed at PPD Laboratories in Richmond, Virginia. The lower limit of quantification for olezarsen was 0.1303 ng/mL. Pharmacodynamic assessments were based on venous blood samples and measured effects of olezarsen on apoC-III, fasting serum triglycerides, and other lipids/lipoproteins including apolipoprotein B-100 (apoB), total cholesterol, high-density lipoprotein cholesterol (HDL-C), low-density lipoprotein cholesterol (LDL-C), and very low-density lipoprotein cholesterol (VLDL-C). These biomarkers and triglycerides were measured at GenX Laboratories (Los Angeles, CA, USA) with commercial assays.

### Statistical analysis

Demographics, baseline characteristics, and safety data were all summarized using descriptive statistics by treatment group and visit, as appropriate. P-values were reported, if applicable. Unless otherwise stated, all statistical analysis was conducted using 2-sided tests with 5% type I error rates. Due to the low number of participants randomized to placebo in the single-ascending–dose cohorts, these participants were analyzed as a separate pooled placebo group. For the pharmacodynamic endpoints of change and percentage change from baseline in triglycerides and apoC-III, levels were compared between olezarsen treatments and pooled placebo using 1-way analysis of variance (ANOVA). Baseline levels were defined as the average of the predose result closest to Day 1 and on Day 1; nonfasting results were not used for baseline calculations. A power model (pharmacokinetic parameter = α Dose^β^) was used to assess the dose proportionality of olezarsen exposure (eg, area under the curve [AUC] from time 0 to the last quantifiable concentration [AUC_last_], AUC from time 0 to infinity [AUC_inf_], maximum observed concentration [C_max_]). If the power model was determined to be inappropriate for dose-proportionality assessment, then the log-transformed dose-normalized data were analyzed using an ANOVA with dose as the classification factor. Within the ANOVA framework, pairwise comparisons among doses were made. In cases where 1 patient’s data was inconsistent with previous data and considered to be an outlier value based on the most extreme value across all the timepoints, an analysis was performed without the inconsistent data. There was no formal statistical hypothesis testing. The planned sample size was consistent with typical phase 1 study sample sizes and deemed sufficient for safety and tolerability assessments. Placebo-corrected values for the olezarsen treated groups were presented as the least square mean change from baseline value minus the least square mean change from baseline value in the pooled-placebo group.

## Results

### Participant disposition and baseline demographics

Between March 1, 2019, and March 3, 2021, 20 participants were treated in the single-ascending–dose part of the study, and 8 participants were treated in the multiple-dose part. All but 1 participant completed the study; 1 participant in the olezarsen 90-mg single-dose cohort withdrew at his own request during the post-treatment follow-up period.

Demographics and baseline characteristics are summarized in Table 1. Participants were between the ages of 27 and 62 years in the single-dose cohorts, and between 21 and 55 years in the multiple-dose cohort. Mean body mass index (BMI) was 23.9 kg/m^2^ in the single-dose cohorts and 23.4 kg/m^2^ in the multiple-dose cohort. Mean apoC-III baseline levels were 14.3 mg/dL and 13.2 mg/dL in the single-dose and multiple-dose cohorts, respectively. Mean triglyceride baseline levels were 159.7 mg/dL and 159.8 mg/dL in the single-dose and multiple-dose cohorts, respectively.

**Table 1:** Participant demographics and baseline characteristics

Single Ascending Dose Cohorts							
Parameter	Placebo *n* = 4		Olezarsen n = 16			Overall N = 20	
		30 mg n = 5		60 mg n = 5	90 mg n = 6		
Age, years, mean (SD)	49.8 (11.95)	37.8 (11.95)		45.6 (10.36)	41.7 (10.42)		43.3 (11.08)
Sex, n (%)							
Male	3 (75.0)	4 (80.0)		4 (80.0)	4 (66.7)		15 (75.0)
Female	1 (25.0)	1 (20.0)		1 (20.0)	2 (33.3)		5 (25.0)
Asian race, n (%)	4 (100.0)	5 (100.0)		5 (100.00)	6 (100.0)		20 (100.0)
Weight, kg, mean (SD)	66.8 (5.15)	64.1 (7.25)		66.3 (9.78)	70.2 (11.01)		67.0 (8.54)
BMI, kg/m^2^, mean (SD)	24.2 (1.34)	23.3 (2.08)		23.6 (1.77)	24.6 (2.58)		23.9 (1.97)
ApoC-III, mg/dL, mean (SD)	14.5 (4.70)	11.7 (1.88)		14.2 (5.83)	16.4 (6.95)		14.3 (5.22)
ApoB-100, mg/dL, mean (SD)	92.5 (14.96)	94.5 (34.26)		93.9 (25.88)	94.8 (28.01)		94.0 (25.11)
Total cholesterol, mg/dL, mean (SD)	200.9 (26.95)	196.6 (45.27)		199.4 (36.72)	215.3 (59.18)		203.8 (42.59)
HDL cholesterol, mg/dL, mean (SD)	52.9 (3.40)	57.4 (12.95)		53.5 (11.36)	56.1 (15.10)		55.1 (11.30)
LDL cholesterol, mg/dL, mean (SD)	124.8 (19.62)	117.0 (48.12)		117.7 (37.27)	125.3 (45.29)		121.2 (37.37)
VLDL cholesterol, mg/dL, mean (SD)	30.5 (20.17)	21.2 (7.97)		30.3 (18.71)	43.0 (24.42)		31.9 (19.45)
Triglycerides, mg/dL, mean (SD)	153.6 (101.49)	106.1 (39.59)		152.0 (93.13)	214.8 (121.64)		159.7 (97.03)
CRP, mg/dL, mean (SD)	0.10 (0.029)	0.13 (0.220)		0.05 (0.041)	0.07 (0.061)		0.09 (0.112)
Multiple Dose Cohort							
Parameter	Placebo n = 2		Olezarsen 60 mg n = 6			Overall N = 8	
Age, years, mean (SD)	32.5 (2.12)		41.8 (13.89)			39.5 (12.54)	
Gender, n (%)							
Male	2 (100.0)		3 (50.0)			5 (62.5)	
Female	0		3 (50.0)			3 (37.5)	
Asian race, n (%)	2 (100.0)		6 (100.0)			8 (100.0)	
Weight, kg, mean (SD)	67.0 (7.14)		63.5 (9.07)			64.3 (8.29)	
BMI, kg/m^2^, mean (SD)	22.3 (2.90)		23.7 (4.18)			23.4 (3.76)	
ApoC-III, mg/dL, mean (SD)	11.0 (1.24)		13.9 (4.44)			13.2 (4.02)	
Apolipoprotein B-100, mg/dL, mean(SD)	68.3 (12.37)		98.3 (17.72)			90.8 (20.98)	
Total cholesterol, mg/dL, mean (SD)	182.8 (17.32)		226.0 (33.42)			215.2 (35.23)	
HDL cholesterol, mg/dL, mean (SD)	68.8 (16.62)		53.5 (11.55)			57.3 (13.59)	
LDL cholesterol, mg/dL, mean (SD)	108.0 (47.38)		149.3 (27.76)			139.0 (35.18)	
VLDL cholesterol, mg/dL, mean (SD)	16.5 (2.83)		37.2 (16.79)			32.0 (17.15)	
Triglycerides, mg/dL, mean (SD)	83.0 (14.85)		185.3 (83.56)			159.8 (85.22)	
CRP, mg/dL, mean (SD)	0.22 (0.219)		0.14 (0.158)			0.16 (0.161)	

*ApoB* apolipoprotein B, *ApoC* apolipoprotein C, *BMI* body mass index, *CRP* C-reactive protein, *HDL* high-density lipoprotein, *LDL* low-density lipoprotein, *SD* standard deviation, *VLDL* very low-density lipoprotein

### Primary endpoint: safety results

A summary of safety results is shown in Table 2. Of the 20 participants in the single-ascending–dose cohorts, 8 (40.0%) reported treatment-emergent AEs (TEAEs). Across single- and multiple-dose cohorts, no serious or severe events were reported; all TEAEs were mild in severity. No TEAEs led to study discontinuation and no deaths were reported. Events reported in > 1 participant across dosing cohorts were chills (*n* = 2), diarrhea (*n* = 2), and headache (*n* = 2). Injection site reactions were minimal and only reported in the 90-mg olezarsen group; injection site bruising (*n* = 1) and injection site pruritus (*n* = 1). No clinically significant abnormalities were identified in laboratory results including metabolic panel, liver and kidney function tests, platelet count or high sensitivity C-reactive protein, vital signs, ECG assessments, or physical examinations. HgA1c was not measured in this study.

**Table 2:** Summary of treatment-emergent adverse events

	Single Ascending Dose cohorts				Multiple Dose cohort	
n (%)	Placebo *n* = 4	Olezarsen 30 mg *n* = 5	Olezarsen 60 mg *n* = 5	Olezarsen 90 mg n = 6	Placebo *n* = 2	Olezarsen 60 mg *n* = 6
Any TEAE	2 (50.0)	2 (40.0)	2 (40.0)	2 (33.3)	0	2 (33.3)
Related TEAE	1 (25.0)	1 (20.0)	0	1 (16.7)	0	2 (33.3)
Chills	0	0	1 (20.0)	0	0	1 (16.7)
Diarrhea	1 (25.0)	1 (20.0)	0	0	0	0
Headache	0	0	0	0	0	2 (25.0)

*TEAE* treatment-emergent adverse event

### Secondary endpoint: Plasma concentration and pharmacokinetics of olezarsen

Plasma concentrations of olezarsen peaked approximately 3 h following 30 mg and 60 mg single doses, and approximately 4 h following 90 mg single doses. Plasma concentrations were quantifiable across all doses in all participants up to 336 h (15 days) post-dose. Following 30 mg single doses, the latest quantifiable concentrations were observed up to 696 h (30 days) post-dose; for 60 and 90 mg single doses, quantifiable concentrations were observed up to 3576 h (150 days) post-dose. Olezarsen plasma concentrations declined in a biexponential manner after C_max_ was reached following single doses. The majority of plasma exposure occurred within the first 24 h post-dose (Supplementary Fig. 2A).

In the single-dose assessment, olezarsen was eliminated from plasma with a geometric mean terminal half-life between 197 and 659 h (8.2–27.5 days). Increases to C_max_, AUC_last_, and AUC_inf_ were slightly greater than dose proportional. The area under the plasma concentration–time curve from 0 to 24 h (AUC_(0-24h)_) geometric mean (CV%) was 1,270 (30.3) ng*h/mL, 3,020 (37.8) ng*h/mL, and 5,590 (34.3) ng*h/mL for 30, 60, and 90 mg doses, respectively. The volume of distribution following single doses of olezarsen was indicative of extensive tissue biodistribution (range, 7,050–1,2800 L). The fraction of olezarsen excreted over 24 h following a single dose suggested negligible renal elimination (range, 0.0264%–0.0741%). Renal clearance over the first 24 h increased with dose and ranged from 6.24 to 12.0 mL/h.

Following multiple doses of 60 mg olezarsen (every 4 weeks for 4 doses), plasma concentrations peaked at approximately 2 h, were quantifiable in all participants up to Day 141, and declined in a biexponential manner (Supplementary Fig. 2B, C). The AUC_(0-24h)_ geometric means (CV%) were 3,500 (32.8) ng*h/mL and 4,190 (54.5) ng*h/mL following the Day 1 and Day 85 olezarsen 60 mg doses, respectively. The mean terminal half-life of olezarsen was 738 h (30.75 days), and accumulation of olezarsen appeared minimal following the multiple-dose regimen. The geometric mean clearance_(0-24h)_/F was 17.1 L/h following Day 1 single-dose olezarsen administration and 14.3 L/h following Day 85 multiple-dose administration. On Day 85, the volume of distribution was approximately 11,700 L, indicative of extensive tissue biodistribution. The fractions of urinary excretion following doses on Day 1 (0.0772%) and Day 85 (0.171%) were suggestive of negligible renal elimination. Following administration on Day 1, renal clearance over the first 24 h was 12.0 mL/h. On Day 85, renal clearance over the first 24 h after administration was 20.8 mL/h.

### Exploratory endpoints: Effect of olezarsen on levels of apoC-III and triglycerides

#### ApoC-III levels

**Fig. 1:**
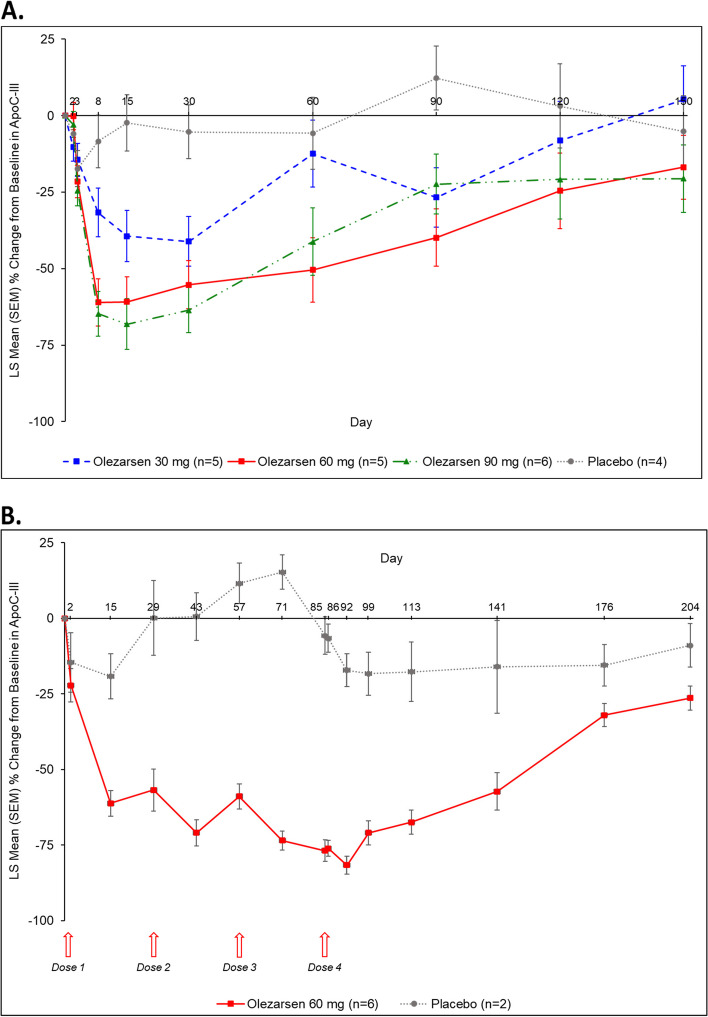
Percentage change from baseline in apoC-III in single-ascending–dose cohort (**A**) and multiple-dose cohort (**B**). apoC-III, apolipoprotein C-III; LS, least squares; SEM, standard error of the mean

Following single-dose administration of olezarsen at doses of 30, 60, or 90 mg statistically significant placebo-corrected decreases in apoC-III levels from baseline were observed beginning from Day 2, reached nadir at Day 30 in the 30-mg group, Day 8 in the 60-mg group, and Day 15 in the 90-mg group, and plateaued thereafter up to Day 120 for the 30-mg group and Day 150 for the 60-mg and 90-mg groups (Fig. 1A). The greatest percentage reduction in apoC-III from baseline was 39.4% at Day 30 in the 30 mg dose, 60.8% at Day 15 in the 60 mg dose, and 68.1% at Day 15 at the 90 mg dose. The placebo group had a 2.3% increase at Day 15. At Day 15, which represents the maximal effect of the two higher single doses, the placebo-corrected least square mean percentage reductions from baseline (*P*-value) were − 37.1% (95% confidence interval [CI]: − 63.9 to − 10.2, *P* = 0.0103), 30 mg; − 58.5% (95% CI: − 84.8 to − 32.2, *P* = 0.0003), 60 mg; and − 65.8% (95% CI: − 92.1 to − 39.5, *P* = 0.0001), 90 mg. Significant differences between olezarsen and placebo were observed as late as 90 days following single-dose administration. At Day 90, the placebo-corrected least square mean percentage reductions from baseline were − 39.0% (95% CI: − 69.6 to − 8.3, *P* = 0.0164), 30 mg; − 52.1 (95% CI: − 82.1 to − 22.0, *P* = 0.0023), 60 mg; and − 34.6 (95% CI: − 65.4 to − 3.8, *P* = 0.0303), 90 mg.

In the multiple dose cohort, following dosing of olezarsen 60 mg every 4 weeks, consistent decreases in apoC-III levels were observed in participants. Decreases from baseline and differences from placebo were observed from Day 2 to Day 204 (Fig. 1B). The greatest percentage reduction in apoC-III was at 92 days with − 81.6% reduction vs − 17.2% reduction in placebo.

#### Triglyceride levels

**Fig. 2:**
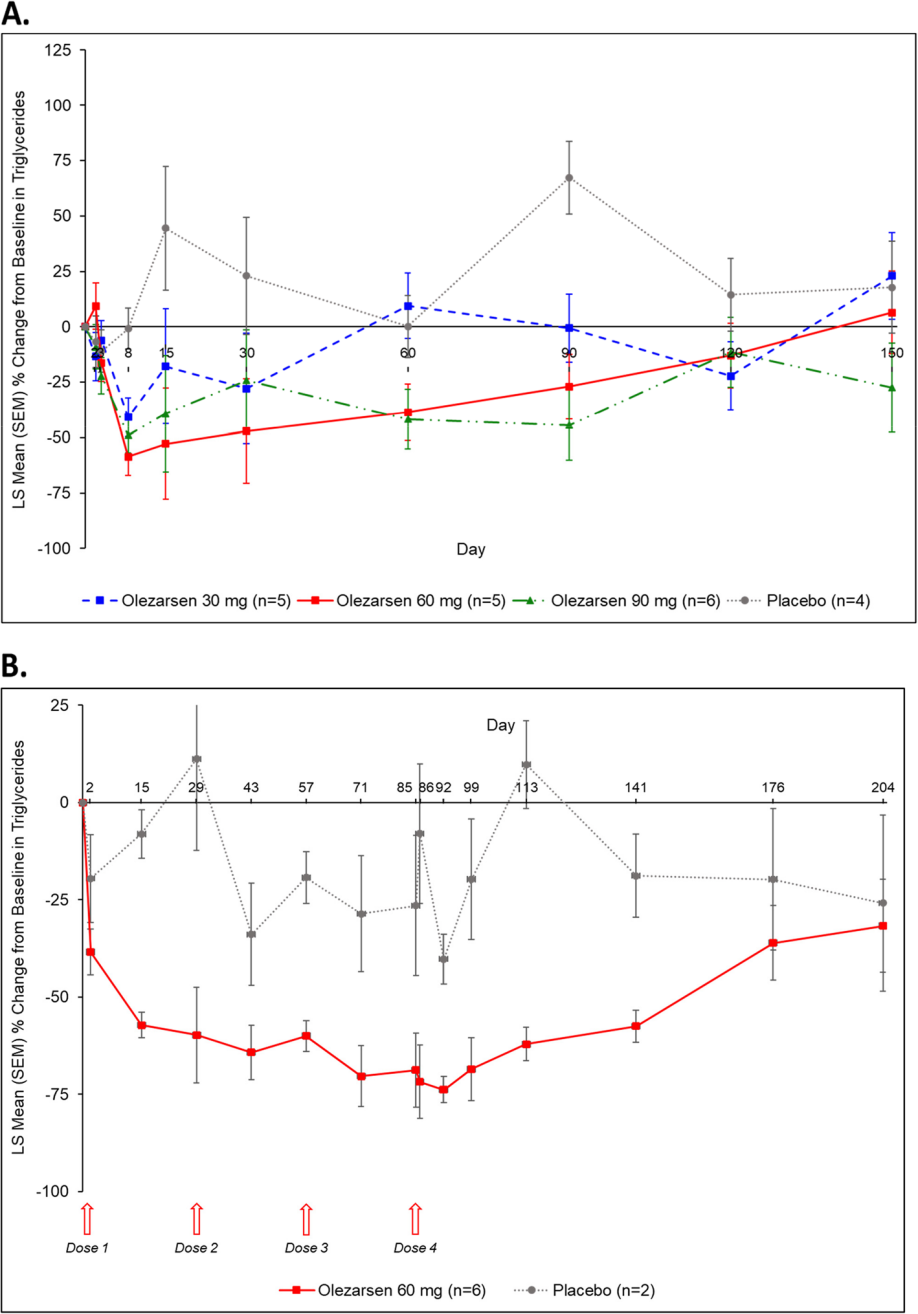
Percentage change from baseline triglycerides^a^ in the single-dose cohort (**A**) and multiple-dose cohort (**B**). LS, least squares; SEM, standard error of the mean. ^a^One subject was excluded from this analysis in the 30 mg olezarsen cohort with a baseline triglyceride level of 93 mg/dL and a Day 60 triglyceride level of 753 mg/dL (809.68% change)

Following single-dose administration of olezarsen at doses of 30, 60, or 90 mg, statistically significant placebo-corrected decreases in triglyceride levels were observed as early as Day 8, with placebo-corrected least square mean percentage reductions from baseline of − 39.9% (95% CI: − 66.9 to − 12.9, *P* = 0.0065), 30 mg; − 57.7% (95% CI: − 84.2 to − 31.2, *P* = 0.0003), 60 mg; and − 48.0 (95% CI: − 74.2 to − 21.7, *P* = 0.0015), 90 mg (Fig. 2A). The greatest percentage reduction in triglycerides from baseline at Day 15 were − 17.8% in the 30 mg dose, − 52.7% in the 60 kg dose, and − 39.2% in the 90 mg dose, vs 44.5% increases in placebo. At Day 15, the placebo-corrected least square mean percentage reductions from baseline were − 62.4% (95% CI: − 144.1 to 19.4, *P* = 0.1239), 30 mg; − 97.2% (95% CI: − 177.6 to − 16.9, *P* = 0.0211), 60 mg; and − 83.8% (95% CI: − 165.9 to − 1.6, *P* = 0.0462), 90 mg. At Day 90, the placebo-corrected least square mean percentage reductions from baseline were − 67.8% (95% CI: − 115.7 to − 20.0, *P* = 0.0088), 30 mg; − 94.3% (95% CI: − 141.3 to − 47.3, *P* = 0.0007), 60 mg; and − 111.7% (95% CI: − 160.9 to − 62.6, *P* = 0.0002), 90 mg.

Consistent decreases from baseline in triglyceride levels were observed in the multiple dose cohort, with decreases seen as early as Day 2 and as late as Day 204 (Fig. 2B). The greatest percentage reduction from baseline in triglycerides was at 92 days with -73.8% reduction in olezarsen vs -40.8% reduction in placebo. Compared to placebo, the least mean squares placebo-corrected percentage reduction ranged from − 71.0% (95% CI: − 143.5% to 1.5%, *P* = 0.0534) at Day 29, − 42.3% (95% CI: − 98.0% to 13.5%, *P* = 0.1088) at Day 85, − 72.0% (95% CI: − 106.2% to − 37.7%, *P* = 0.0043) at Day 113, − 16.4% (95% CI: − 72.5% to 39.8%, *P* = 0.4881) at Day 176, and − 5.8% (95% CI: − 75.8% to 64.1%, *P* = 0.8385) on Day 204.

### Additional lipid biomarkers

Following single-dose administration of olezarsen, changes from baseline in apoB, cholesterol, HDL-C, VLDL-C, and LDL-C were transient and not dose-dependent (Supplementary Table 2).

**Fig. 3:**
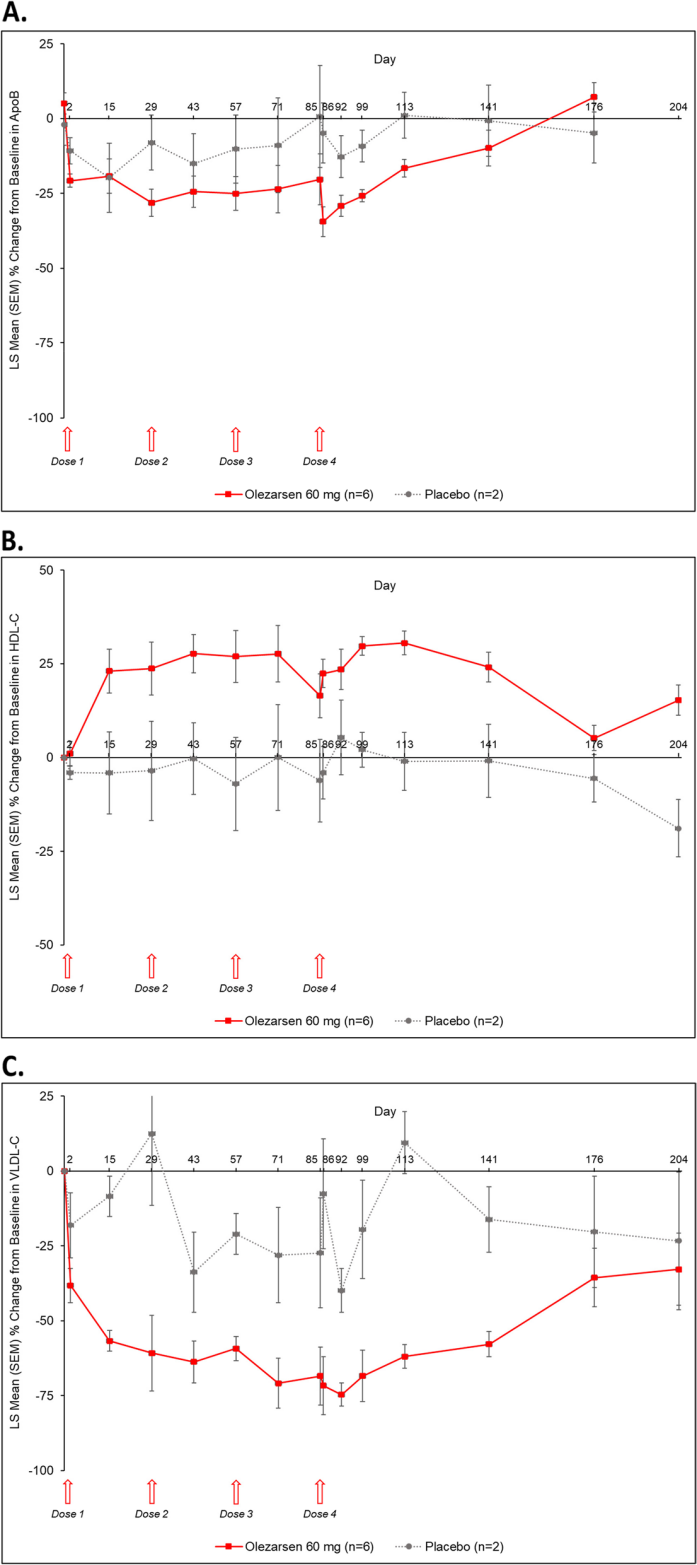
Percentage change from baseline in apoB (**A**), HDL-C (**B**), and VLDL-C (**C**) in the multiple-dose cohort. apoB, apolipoprotein B; HDL-C, high-density lipoprotein cholesterol; LS, least squares; SEM, standard error of the mean; VLDL-C, very-low density lipoprotein cholesterol

Following the multiple dose regimen, changes from baseline in VLDL-C (decrease), apoB (decrease), and HDL-C (increase) were observed; significant differences from placebo were observed in VLDL-C (Day 15 by − 48.28%, Day 57 by − 38.22%, Day 86 by − 64.05%, Day 92 by − 34.76%, Day 113 by − 71.43%, Day 141 by − 41.64%), apoB by − 16.56% (Day 113), and HDL-C (Day 86 by 26.49%, Day 99 by 27.67%, Day 113 by 31.63%, Day 204 by 34.17%) (Fig. 3).

## Discussion

The results of this study expand the evidence base on the efficacy and safety of the GalNAc_3_-conjugated ASO olezarsen in Japanese American subjects. Olezarsen was safe and well tolerated with no serious treatment emergent adverse events, drug related treatment discontinuations, and no platelet, liver or renal abnormalities. The pharmacokinetic profile was typical of GalNAc_3_-conjugated ASOs. The exploratory efficacy data demonstrated significant apoC-III and triglyceride reductions at most doses studied. Specifically, in the multiple dose cohort, the greatest percentage reduction in apoC-III and triglycerides from baseline were at Day 92 with − 81.6% and − 73.8% reduction. The responses to olezarsen in Japanese American subjects were similar to the responses observed in mainly White subjects at the same doses and similar timepoints [9, 10].

In this study, olezarsen was generally safe and well-tolerated with no serious or severe TEAEs. All TEAEs reported were judged to be mild. No clinically significant abnormalities were identified in laboratory results (including high sensitivity C-reactive protein), vital signs, ECG assessments, or physical examinations. These safety results are similar to those of two larger, longer-term (6–12 months) placebo-controlled phase 2 studies in primarily White patients, where the incidences of any AEs with olezarsen occurred at a similar frequency as placebo [10, 17]. These findings are consistent with the broader experience of GalNAc-modified ASOs derived from an integrated safety assessment of data from 7 phase 2 studies [22].

The pharmacokinetic profile of olezarsen is consistent with other GalNAc_3_-conjugated ASOs, which have similar time to maximal concentration and elimination half-lives [23]. In general, the observed C_max_ and AUCs of the GalNAc_3_-conjugated ASOs are lower than those of the non-GalNAc_3_-conjugated parent compounds [23]. The data demonstrate that the majority of plasma exposure to olezarsen occurs within the first 24 h post-dose (AUC_(0_–_24)_ accounts for 80 to 90% of AUC_last_) for Japanese Americans, with negligible renal elimination (< 0.1% of the dose). Plasma clearance of olezarsen in Japanese Americans was similar when administered as single or multiple doses. In a prior phase 1/2 study of olezarsen [9], the same doses of olezarsen (among others) were administered to primarily White subjects. When the pharmacokinetic parameters of the 2 studies were compared, no remarkable differences between the Japanese American and White subjects were identified A similar study of another GalNAc_3_-conjugated ASO, pelacarsen, reported a pattern of increased C_max_ and AUC in some dose cohorts, which was further analyzed and attributed to lower body weight and/or BMI in the Japanese American population [24]. However, the magnitude of change observed in these parameters would not generally require dose adjustment based on analysis of the overall safety and efficacy.

Significant reductions in apoC-III and fasting triglyceride levels were observed in this study following single-dose administration. The time courses of these reductions were similar for apoC-III and triglycerides and both were similar to the results of the phase 1/2 study in healthy White subjects with similar baseline concentrations of apoC-III and triglycerides per cohort (8.5–14.8 mg/dL and 127–245 mg/dL, respectively) [9]. In the phase 1/2 study at Day 15 placebo-corrected reductions in apoC-III for single doses of 30, 60, and 90 mg olezarsen were − 57%, − 90%, and − 101%, whereas placebo-corrected reductions in triglyceride levels were − 33%, − 65%, and − 109% [9].

Following multiple-dose administration of 4 doses of 60 mg olezarsen over 3 months, significant placebo-corrected reductions in apoC-III and triglycerides were observed beginning at Day 15. The placebo-corrected reduction in apoC-III nadired at Day 43, with continued significance through Day 141. Similarly, the placebo-corrected reduction in fasting triglycerides nadired at Day 29 but did not reach significance potentially due to variability at several time points (Day 29, 43, 71, 85, and 99), last reaching significance at Day 141.

The variability in apoC-III and triglycerides compared to other lipid biomarkers, noted in placebo-treated patients, is consistent with previously observed studies [25]. A main determinant of this is the dominant contribution of dietary fat (triglycerides) intake on plasma triglycerides, despite using fasting values [26, 27]. Therefore, the efficacy endpoints are dependent on both dietary intake as well as the effect of olezarsen. The changes in triglyceride levels over time observed in the study, particularly in the small number of placebo-treated patients, with few patients per cohort, emphasizes the variability in fasting triglyceride measurements. For example, in a single patient, > 800% increase from baseline to Day 60 in triglycerides was observed. While the apoC-III and triglyceride measures followed generally the same trajectories, these data demonstrate lower variability in apoC-III measurements, which likely are less affected by dietary lipid uptake.

Olezarsen has been shown previously to reduce TRL, with nuclear magnetic resonance spectroscopy documentation of large percentage reductions in medium- and large-size particles [28]. As a result of these broader effects of apoC-III lowering, other lipid measures were observed to be altered by olezarsen, with reductions noted in VLDL, apoB, and non–HDL-C, and an increase in HDL-C [9, 10]. In the study, significant reductions in VLDL and apoB were observed and increases in HDL-C were noted. Plasma triglycerides in Japanese patients with diabetes are associated with risk for coronary heart disease, suggesting that risk from high apoC-III/triglyceride levels may be the same for Japanese descendants as for descendants of other ancestral groups [29].

These findings in the Japanese American subjects, where 30, 60, and 90 mg doses were studied, support the dosing regimens chosen in the phase 3 trial program for olezarsen. Balance (FCS) [16], Bridge (HTG) [17], CORE and CORE-2 (SHTG; ongoing), and ESSENCE (HTG; ongoing) all study the efficacy and safety of 50 and 80 mg doses of olezarsen. As shown in the multiple-dosing group, apoC-III and triglyceride reductions may increase over time on therapy.

### Limitations of study

Due to the small sample size, it may be difficult to generalize the results. Although the study procedures included strict fasting and dietary restriction guidelines, it is possible that the study subjects did not adhere completely to the guidelines, causing variability in lipid measurements. Additional considerations for the interpretation and implication of these results include a general lack of data from other minorities, different countries, and comparisons between people of different backgrounds. Evaluation of efficacy and of long-term safety requires longer, larger, and controlled studies. It is known that aerobic exercise may affect apoC-III and triglyceride levels, [30] but formal studies of physical activity were not performed in this study. Finally, study subjects were expected to follow their usual dietary and activity habits during the study period, therefore the concomitant effect of a low-fat diet needs to be evaluated in larger studies in more targeted populations such as patients with severe hypertriglyceridemia.

## Conclusions

In summary, single- and multiple-dose administration of olezarsen was considered safe and well-tolerated in the Japanese American population studied. Doses of 60 mg every 4 weeks reduced apoC-III and triglyceride levels and support the use of similar or somewhat higher doses in the olezarsen development program.

## Supplementary Information

Supplementary Material 1. 

## Data Availability

Collaboration may contact the corresponding author. Data requests from qualified researchers will be considered by Ionis Pharmaceuticals once all three of the following criteria are met: (1) 12 months from marketing approval of the study drug in both the United States and European Union; (2) 18 months from conclusion of the study; and (3) 6 months from publication of this article. For additional information, visit https://vivli.org/ourmember/ionis/.

## Abbreviations

AE: Adverse event
ANOVA: Analysis of variance
apoB: Apolipoprotein B-100
apoC-III: Apolipoprotein C-III
ASO: Antisense oligonucleotide
AUC: Area under the curve
AUC_inf_: Area under the curve from time 0 to infinity
AUC_last_: Area under the curve from time 0 to the last quantifiable concentration
AUC_(0-24h)_: Area under the plasma concentration–time curve from 0 to 24 h
BMI: Body mass index
CI: Confidence interval
C_max_: Maximum observed concentration
ECG: Electrocardiogram
ECL: Electrochemiluminescence
FCS: Familial chylomicronemia syndrome
HDL-C: High-density lipoprotein cholesterol
LDL: Low-density lipoprotein
LDL-C: Low-density lipoprotein cholesterol
MD: Multiple doses
SAD: Single-ascending doses
SHTG: Severe hypertriglyceridemia
TEAE: Treatment-emergent adverse event
TRL: Triglyceride-rich lipoprotein
VLDL: Very low-density lipoprotein
VLDL-C: Very low-density lipoprotein cholesterol
