# Supplementary material for: Efficacy and safety of olezarsen in lowering apolipoprotein C-III and triglycerides in healthy Japanese Americans
Source: Lipids Health Dis. 2024 Oct 3;23:329. doi: 10.1186/s12944-024-02297-5 (PMC11448427; doi:10.1186/s12944-024-02297-5)
Supplement: Supplementary file 1 — Supplementary Material 1. [file 12944_2024_2297_MOESM1_ESM.docx]

# Supplemental material

## Supplementary Table 1. Randomization scheme.

| Single Ascending Dose | | | |
| --- | --- | --- | --- |
| Cohort No. | Olezarsen or Placebo | Number of Participants | Active:Placebo |
| 1 | 30 mg | 6 | 5:1 |
| 2 | 60 mg | 6 | 5:1 |
| 3 | 90 mg | 8 | 6:2 |
| Multiple Dose | | | |
| Cohort No. | Olezarsen or Placebo | Number of Participants | Active:Placebo |
| 4 | 60 mg | 8 | 6:2 |

**Supplementary Table 2.** Changes in lipid variables at Day 15 in the single dose cohorts.

|  | **LS Mean (SEM) % Change from Baseline** | | | | ***p-*value vs placebo** |
| --- | --- | --- | --- | --- | --- |
| **Variable** | Olezarsen 30 mg  (n=5) | Olezarsen 60 mg  (n=5) | Olezarsen 90 mg  (n=6) | Placebo  (n=4) |  |
| **VLDL-C**^a^ | −18.0 (26.4) | −51.4 (25.4) | −39.0 (26.6) | 45.20 (28.4) | 30 mg; *p*=0.124  60 mg; *p*=0.0235  90 mg; *p*=0.048 |
| **ApoB**^b^ | −4.4 (4.9) | −10.3 (4.9) | −16.0 (4.9) | −3.7 (5.5) | 30 mg; *p*=0.91  60 mg; *p*=0.38  90 mg; *p*=0.12 |
| **HDL-C** | 6.0 (4.3) | 25.50 (4.3) | 23.9 (4.3) | 5.6 (4.8) | 30 mg; *p*=0.9481  60 mg; *p*=0.0078  90 mg; *p*=0.0126 |
| **Total Cholesterol** | −0.28 (4.2) | −0.35 (4.239) | −6.4 (4.2) | −1.42 (4.7) | 30 mg; *p*=0.86  60 mg; *p*=0.87  90 mg; *p*=0.45 |
| **LDL-C** | 3.8 (8.7) | −0.37 (8.7) | −8.9 (8.8) | 0.07 (9.8) | 30 mg; *p*=0.78  60 mg; *p*=0.97  90 mg; *p*=0.51 |

^a^One subject was excluded from the analysis in the 30 mg cohort with a baseline VLDL-C level of 18.5 mg/dL and a Day 60 VLDL-C level of 150.5 mg/dL (813.51 percentage change from baseline).

^b^One subject was excluded from the analysis in the 90 mg cohort with a baseline apoB level of 122.5 mg/dL and a Day 30 apoB level of 1878.5 mg/dL (1533.47 percentage change).

ApoB, Apolipoprotein B; LDL-C, low-density lipoprotein cholesterol; LS, least squares; VLDL-C, very low-density lipoprotein cholesterol; LS, least squares; SEM, standard error of the mean.

## Supplementary Figure 1. Consort diagram.


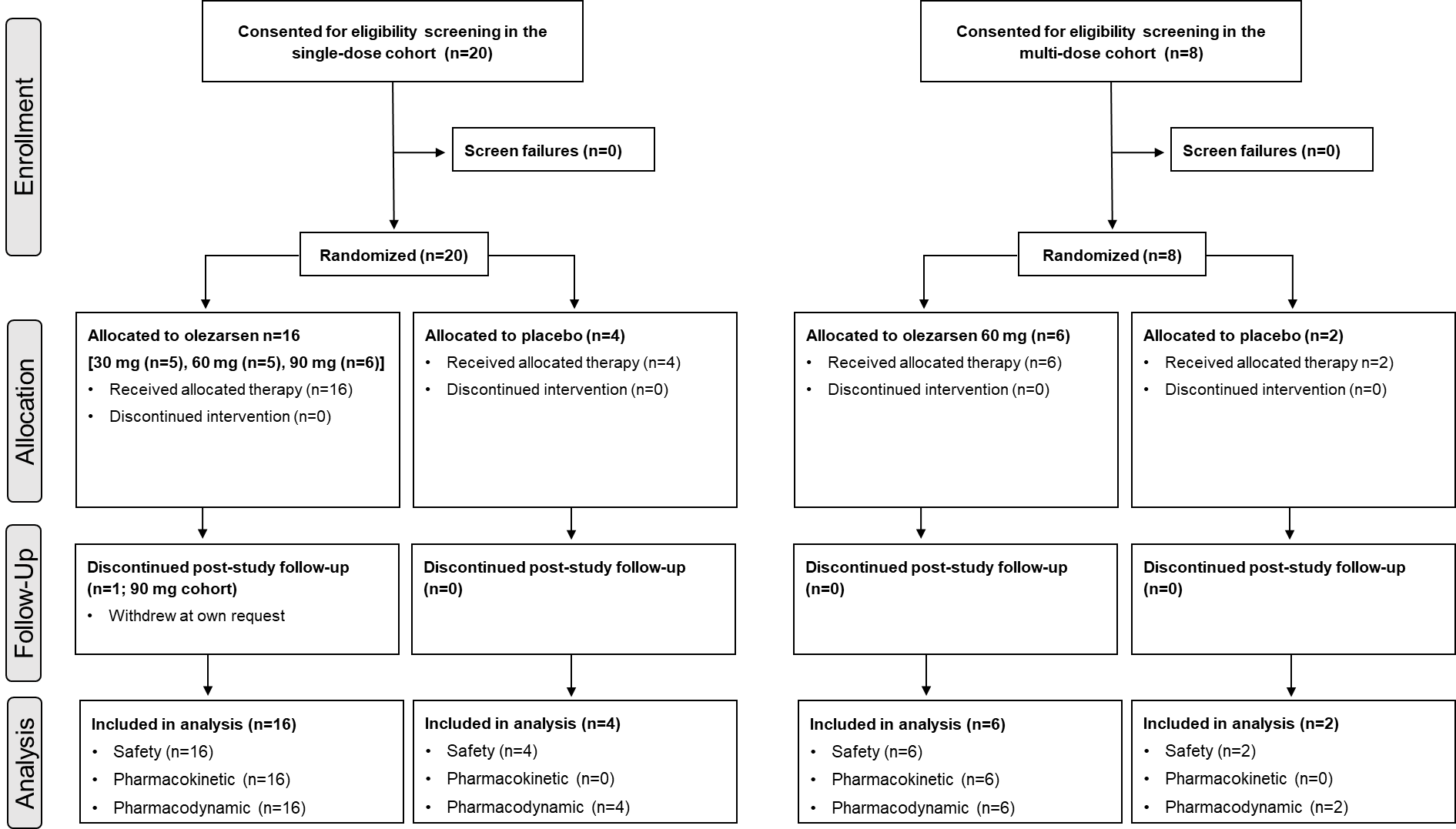


## Supplementary Figure 2. Plasma concentrations of olezarsen in single-ascending (A) and multiple-dose (B and C) cohorts.

**A.**

**
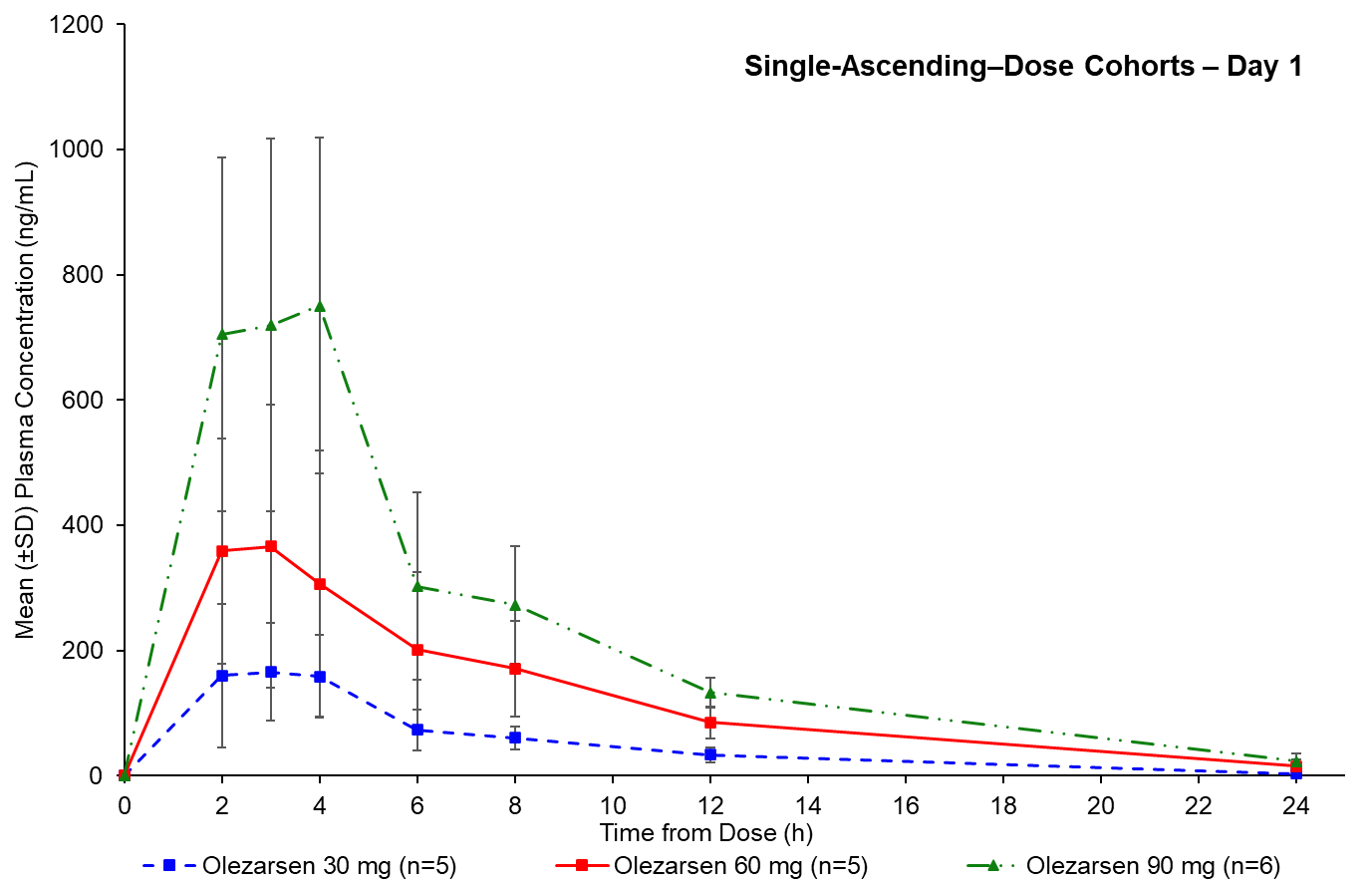
**

**B.**

**
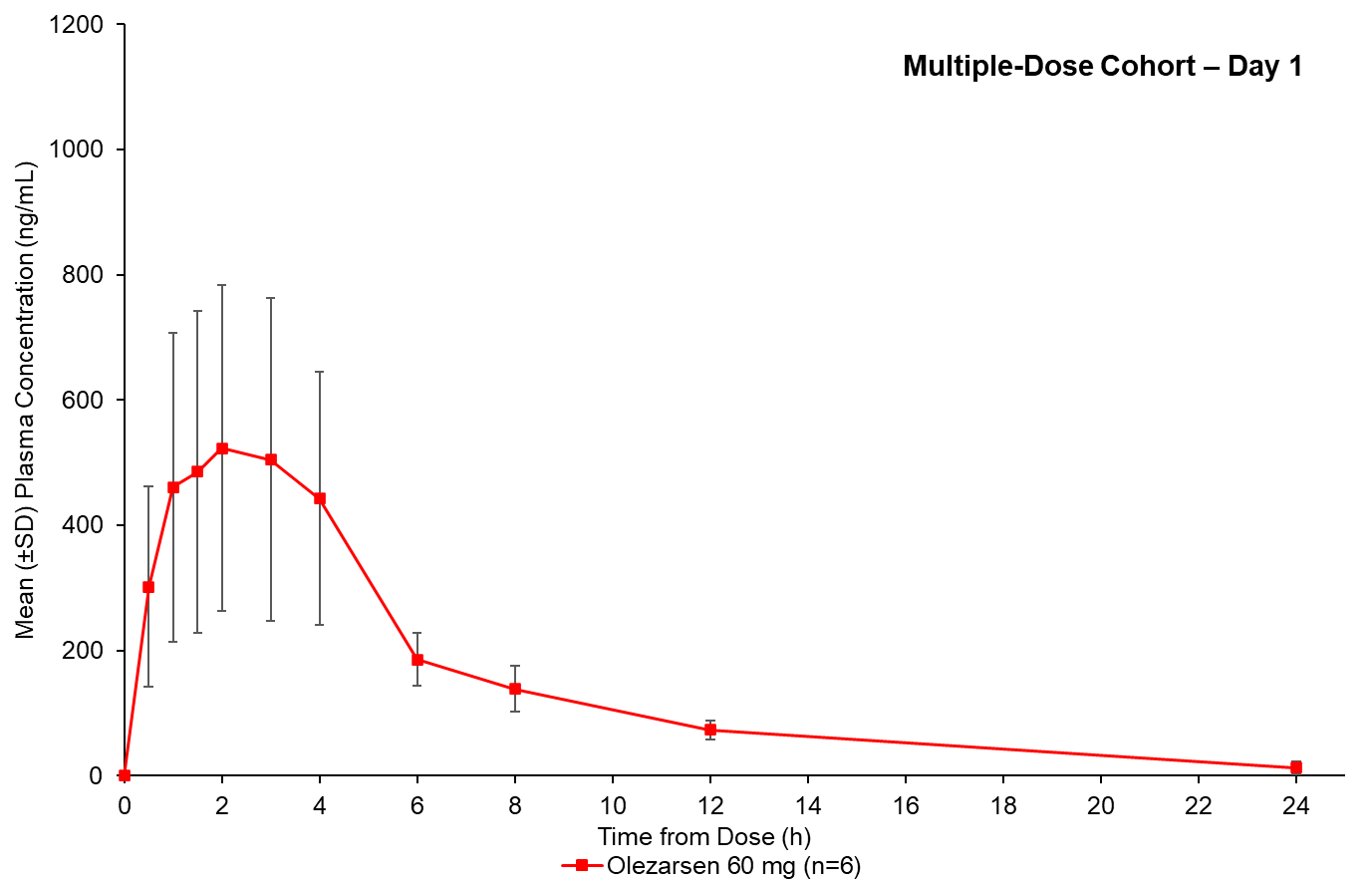
**

**C.**

**
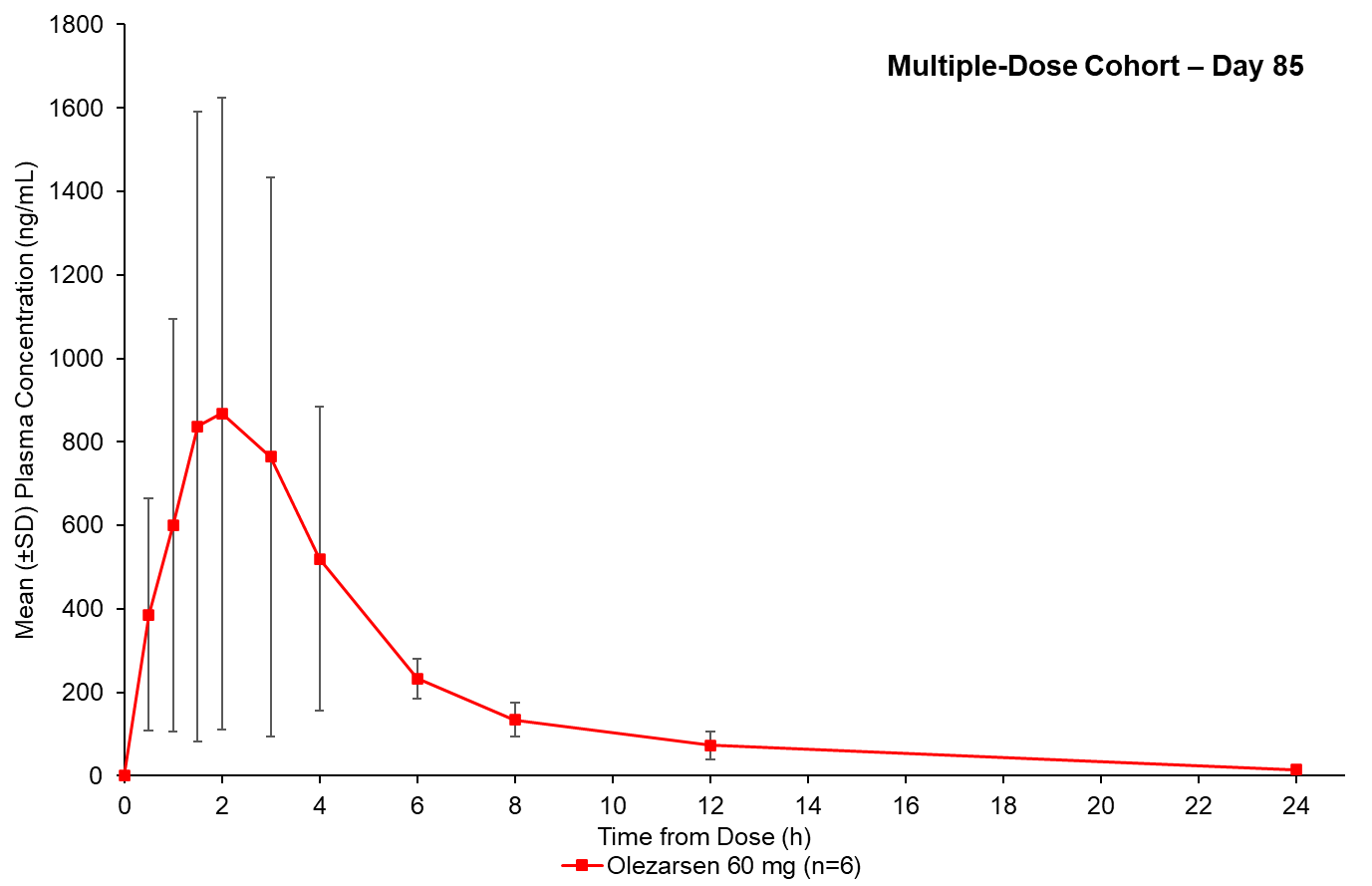
**
